# USING VR FOR NAVIGATION ASSESSMENT IN OLDER ADULTS

**DOI:** 10.1093/geroni/igz038.896

**Published:** 2019-11-08

**Authors:** Scott Moffat

**Affiliations:** Georgia Institute of Technology, Atlanta, Georgia, United States

## Abstract

There has been a long tradition of wayfinding and orienteering studies in humans but these have mostly neglected possible age-related differences in navigation. This field of inquiry is experiencing something of a resurgence of interest due to the development of VR technology which has brought the systematic study of large scale navigation into the laboratory and into the MRI scanning environment. Empirical studies to date identify navigation as an aspect of cognition that is vulnerable to the aging process. Functional and structural neuroimaging studies in humans suggest that age-related changes in the brain’s “navigation circuit” may underlie these behavioral age differences. Older adults also adopt unique spatial strategies and knowledge of these strategy preferences could enlighten both basic science research in spatial cognition and also inform the development of age-specific technological assistance that may extend functional independence of older adults into later life.

